# The Pancreatic and Duodenal Homeobox Protein PDX-1 Regulates the Ductal Specific Keratin 19 through the Degradation of MEIS1 and DNA Binding

**DOI:** 10.1371/journal.pone.0012311

**Published:** 2010-08-19

**Authors:** Johannes von Burstin, Maximilian Reichert, Melanie P. Wescott, Anil K. Rustgi

**Affiliations:** Division of Gastroenterology, Departments of Medicine and Genetics, Abramson Cancer Center, University of Pennsylvania, Philadelphia, Pennsylvania, United States of America; University of Western Ontario, Canada

## Abstract

**Background:**

Pancreas organogenesis is the result of well-orchestrated and balanced activities of transcription factors. The homeobox transcription factor PDX-1 plays a crucial role in the development and function of the pancreas, both in the maintenance of progenitor cells and in determination and maintenance of differentiated endocrine cells. However, the activity of homeobox transcription factors requires coordination with co-factors, such as PBX and MEIS proteins. PBX and MEIS proteins belong to the family of three amino acid loop extension (TALE) homeodomain proteins. In a previous study we found that PDX-1 negatively regulates the transcriptional activity of the ductal specific keratin 19 (*Krt19*). In this study, we investigate the role of different domains of PDX-1 and elucidate the functional interplay of PDX-1 and MEIS1 necessary for *Krt19* regulation.

**Methodology/Principal Findings:**

Here, we demonstrate that PDX-1 exerts a dual manner of regulation of *Krt19* transcriptional activity. Deletion studies highlight that the NH_2_-terminus of PDX-1 is functionally relevant for the down-regulation of *Krt19*, as it is required for DNA binding of PDX-1 to the *Krt19* promoter. Moreover, this effect occurs independently of PBX. Second, we provide insight on how PDX-1 regulates the Hox co-factor MEIS1 post-transcriptionally. We find specific binding of MEIS1 and MEIS2 to the *Krt19* promoter using IP-EMSA, and siRNA mediated silencing of *Meis1*, but not *Meis2*, reduces transcriptional activation of *Krt19* in primary pancreatic ductal cells. Over-expression of PDX-1 leads to a decreased level of MEIS1 protein, and this decrease is prevented by inhibition of the proteasome.

**Conclusions/Significance:**

Taken together, our data provide evidence for a dual mechanism of how PDX-1 negatively regulates *Krt19* ductal specific gene expression. These findings imply that transcription factors may efficiently regulate target gene expression through diverse, non-redundant mechanisms.

## Introduction

The pancreas is a multifunctional organ that comprises an endocrine compartment, which regulates glucose homeostasis mainly through insulin secretion and is responsible for the secretion of other hormones, and an exocrine compartment, where acinar cells produce digestive enzymes that are secreted into the intestine via a network of pancreatic ducts. Each distinct cell type of the pancreas (endocrine, acinar and ductal) originates from a common pool of progenitor cells during development and requires a complex pattern of transcription factors [1] as well as mesenchymal-epithelial interactions [2] for proper lineage specification. In the mouse, dorsal and ventral pancreatic buds start as outgrowths from the foregut endoderm on day E9.5 [3], branch and then fuse at E12 to E13. Functional cells can be observed at day E14.5 [4].

The Pancreatic and Duodenal homeobox protein PDX-1 is critical for pancreatic development. It is expressed in the foregut endoderm at E8.5, prior to the onset of bud formation and embryonic deletion of PDX-1 results in pancreatic agenesis [5]. In addition to its crucial functions during development, PDX-1 is also required for accurate endocrine function by regulating endocrine gene expression [6] and β-cell survival in the adult [7]. Recent three-dimensional cell culture studies have revealed the role of PDX-1 in ductal branching morphogenesis or tubulogenesis, as it appears to be re-expressed in otherwise PDX-1 negative pancreatic ductal cells at the very site of branching [8]. Although PDX-1 has been described as a positive regulator of gene expression in endocrine cells, such as insulin and somatostatin, recent evidence has shown that PDX-1 can be a negative regulator of gene expression in non-endocrine pancreatic cells [9].

Keratins belong to the family of intermediate filament proteins and are critical for proper tissue function and maintenance [10]. The family of keratins consists of 54 genes and can be classified into type I (acidic) or type II (basic to neutral) keratins [11], [12]. Typically, one type I keratin forms a heterodimer with a type II keratin. Keratin 19 (*Krt19*) can be found in various stratified and simple epithelial cells. In the pancreas, its expression is restricted to ductal cells and is absent in acinar or endocrine cells. We have demonstrated previously that *Krt19* expression is regulated through KLF4 and Sp1 transcription factors and that differences in distribution of these factors in the pancreas are responsible for ductal specific expression of *Krt19* [13]. To investigate the potential interplay between PDX-1 and ductal cell morphogenesis, we have studied the impact of PDX-1 on *Krt19* transcriptional activity. In this study, we demonstrated transcriptional repression of *Krt19* by PDX-1. In addition, we identified the Hox co-factor family of MEIS proteins as DNA binding partners on the *Krt19* promoter [9].

MEIS (Myeloid ecotropic viral integration site) proteins are members of the three amino acid loop extension (TALE) homeodomain transcription factors and are required for the proper DNA recognition by and the correct function of Hox transcription factors [14]. *Meis1* was first identified as a common viral integration site in BXH-2 mice, which have nearly a 100% rate of spontaneous leukemia [15]. Thereafter, the genes *Meis2* and *Meis3* were identified with a homology of 83% and 66%, respectively, at the amino acid level. Additionally, several splice variants exist for each gene. Of note, the *Xaenopus laevis* homologs *Xmeis1-1* and *Xmeis1-2* share an amino acid sequence identity of 97% and 100% with MEIS1a, suggesting a highly evolutionarily conserved function [16]. Indeed, *Meis1* is essential for embryonic development as *Meis1* knockout mice die during embryogenesis [17] due to hematopoietic and vasculature defects. However, functional differences between the isoforms are not well understood and require elucidation. Other members of the TALE homeodomain transcription factors are PBX proteins that interact with the FPWMK motif of Hox transcription factors. This is also true for PDX-1 in pancreatic development as mutation of FPWMK to AAGGQ in PDX-1 abrogates PBX binding and results in pancreatic hypoplasia [18]. A similar phenotype is also observed in *Pbx1* knockout mice [19] underscoring the importance of PDX-1/PBX1 interactions for pancreatic development.

Here, we set out (1) to characterize the domains of PDX-1 involved in *Krt19* transcriptional repression, (2) to determine how different *Meis* genes affect *Krt19* transcriptional regulation and (3) to elucidate functional interactions between MEIS proteins and PDX-1. We provide evidence for the critical role of the NH_2_-terminus of PDX-1 in mediating *Krt19* repression. In addition, MEIS1 is identified as the main isoform that is functionally relevant in pancreatic ductal cells. MEIS1 binds directly to the *Krt19* promoter and knockdown of *Meis1* mRNA, but not *Meis2* mRNA, decreases *Krt19* mRNA levels. In addition, we show co-expression of MEIS1 and KRT19 in pancreatic ductal cells *in vivo*. Surprisingly, PDX-1 over-expression leads to post-transcriptional down-regulation of MEIS1 protein, which could be prevented by inhibition of the proteasome, although this may potentially be independent of ubiquitination. Taken together, our data suggest a novel, dual mechanism of PDX-1 mediated regulation of *Krt19* transcriptional activity, one directly through DNA binding and the other through down-regulation of the *Krt19* transcriptional activator MEIS1.

## Materials and Methods

### Animals – Ethics Statement

All procedures involving animals were approved by the Institutional Animal Care and Use Committee of the University of Pennsylvania (Protocol #800502). Six to eight week old C57BL/6 mice were purchased at Charles River Laboratories (Wilmington, MA) and housed according to institutional guidelines for another week before experiments were performed.

### Avidin-Biotin DNA precipitation assay

Avidin-Biotin DNA precipitation assay was essentially performed as previously described [20]. Briefly, a total of 1000 µg whole cell extract of cells transfected with pCMX, pCMX-PDX-1 V5 or pCMX-PDX-1 Δ1-37 V5, was incubated with 2 µg biotinylated double stranded oligonucleotide for 4 hours with constant rotation at 4°C in a total volume of 800 µl immunoprecipitation (IP) buffer (50 mM HEPES, 150 mM NaCl, 1 mM EDTA, 0.5% NP-40, 10% glycerol). After addition of 60 µL equilibrated streptavidin agarose beads (Invitrogen, Carlsbad, CA) incubation was continued for 60 minutes at 4°C on a rotating platform. Beads were collected by centrifugation and washed five times with IP buffer. Precipitated beads were boiled in 2× SDS-sample buffer and proteins were separated by sodium dodecyl sulfate-polyacrylamide gel electrophoresis (SDS-PAGE). V5-epitope was detected by Western blot. Densitometry was performed directly on the Odyssey Infrared Imaging System (LiCor, Lincoln, NE). Intensity of bands detected by V5 in the precipitation assay was normalized to input samples. The following 5′ biotinylated oligonucleotides were used:

*Krt19*: 5′-biotin-GGGTGTGATTTCTAAGGGTGTCAAATTCCTGGAGGT-3′

*Ins1*: 5′-biotin-CTTATTAAGACTATAATAACCCTAAGACTA-3′

Scramble: 5′-biotin-GCCGCCGCCGCCGCCGCCGC-3

For competition assays, unbiotinylated *Krt19* oligonucleotide was added in increasing concentrations (2, 10, 20, 50 and 100 µg), and loading control was performed by Coomassie staining.

### Plasmids

Deletions of PDX-1 were generated with a PCR based technique using pCMX-PDX-1 as a template [9]. Primers were selected to create full length PDX-1 or to delete amino acids (AA) 1–37, 1–109, 1–144, 260–284, and 235–284 and to add the coding sequence for the V5-epitope in frame with the C-terminus. Restriction sites were 5′-XhoI and 3′-SacII. Following PCR amplification, coding sequences were subcloned into pIRES2-EGFP. Subsequently, the coding sequence of PDX-1 was removed from pCMX by restriction with XhoI and BamHI and the V5-tagged inserts were subcloned from the pIRES2-EGFP constructs back to pCMX using XhoI and BamHI. Mutation of the pentapeptide motif of PDX-1 FPWMK→AAGGQ was introduced using Quikchange II XL Site Directed Mutagenesis Kit (Stratagene, La Jolla, CA) according to the manufacturer's instructions. MEIS1a was amplified from pCS2-MEIS1a [9] and the coding sequence for FLAG was added in frame with the C-terminus. For this purpose, NheI was used for 5′-restriction and XhoI for 3′-restriction. The amplicon was then inserted into pcDNA3.1(+) (Invitrogen, Carlsbad, CA). pcDNA-MEIS2b-FLAG and pcDNA-MEIS3-FLAG were provided by Dr. Doris A. Stoffers. The pM1, pM1 KOX-KRAB and pGL4.10 Gal4-TK plasmids were generous gifts of Dr. Frank J. Rauscher III and have been described earlier [21]. PDX-1 and PDX-1 1–37 were fused to the GAL4-DNA-binding domain in pM1 by amplification of PDX-1 or PDX-1 1–37 from the PDX-1 vectors described above, using BamHI and SacII restriction sites. For insertion of 5× Gal4 binding motifs into pKrt19 -1970, unique AgeI and SacII restriction sites were introduced at position −367 bp upstream of the transcriptional start site. Subsequently, the 5xGal4 binding motif was removed from pIDTSMART 5xGal (Integrated DNA Technologies, Coralville, IO) using AgeI and SacII and inserted into pKrt19 -1970. pCMV-HA-Ubiqutin was a generous gift of Dr. J. Alan Diehl and has been described previously [22]. Primer sequences used for cloning, generation of deletions or internal mutations can be found in Table S1.

### Cell culture and reagents

HEK 293T cells were purchased from ATCC (Manassas, VA) and cultured in DMEM (Mediatech, Manassas, VA) supplemented with 10% FBS (Sigma, St. Louis, MO) and 1% (w/v) penicillin/streptomycin (Invitrogen, Calrsbad, CA). MIN6 mouse insulinoma cells were provided by Dr. Doris A. Stoffers and cultured as described previously [7]. Pancreatic ductal cells (PDCs) were isolated and maintained as described [23].

### Transfections, reporter assay, proteasome inhibition and treatment with Trichostatin A (TsA)

For reporter studies, PDCs were plated in 12MW plates at a density of 2×10^4^ cells and allowed to attach for 48 hrs. Cells were then transfected using Lipofectamine 2000 and 0.38 µg of the reporter construct (pGL3-basic, pKrt19 -1970 or pGal4-Krt19 -1970), 0.02 µg pRL-CMV and 1.2 µg pCMX-PDX-1, truncated versions of pCMX-PDX-1 or pM1GAL4-constructs. Fory-eight hours post transfection cells were lysed in 100 µl passive lysis buffer and assayed for luciferase activity using the Dual Luciferase Assay Kit (Promega, Madison, WI). In a subset of experiments, treatment with 0.3 µM TsA (Biomol, now part of ENZO Life Sciences, Plymouth Meeting, PA) was performed 24 hrs. prior to the reporter assay. For co-transfection studies, 5×10^5^ HEK 293T cells/well in a 6MW plate were seeded and transfected 24 hrs. later with 2 µg of each plasmid using Lipofectamine 2000. Inhibition of the proteasome was achieved 48 hrs. post transfection by treating cells with 1 µM Bortezomib (LC Labs, Woburn, MA) or 5 µM MG-132 (Biomol, now part of ENZO Life Sciences, Plymouth Meeting, PA) for indicated times. Small interfering RNAs (siRNAs) against *Meis1* and *Meis2* were purchased from Invitrogen (Carlsbad, CA), the sequences were as follows: si*Meis1* #1 5′-TTGAGGCTGACATTGGCATTCCAGG-3′, si*Meis1* #2 5′-TATGGCTTGAATCATCAAGTTATCC-3′, si*Meis2* #1 5′-AATGTCATGCCAGCCAGCATGGGAT-3′ and si*Meis2* #2 5-TGGGCACCCGTTGTTTCCTCTGTTA-3′. Control siRNA was purchased from Dharmacon, part # D-001206-13-05 (Lafayaette, CO). siRNAs were transfected using the Amaxa Nucleofector and the Amaxa Nucleofection Kit V. 240 pmol siRNA were transfected in 2×10^6^ PDCs in 100 µl Nucleofection solution V. Cells were harvested 72 h post-transfection for RNA isolation.

### Quantitative reverse-transcriptase PCR

RNA was isolated using the RNeasy kit (Quiagen, Maryland, MD) and 1 µg was transcribed into cDNA (Taqman Reverse Transcription Reagents, Applied Biosystems, Branchburg, NJ). RNA was primed with random hexamers. Quantitative analysis was carried out on an ABIPrism 7000 sequence detection system and the amount of target gene was normalized to the endogenous reference as described. [24], [25]. Intron spanning isoform specific primer sequences are listed in Table S2.

### Immunoprecipitation-Electrophoretic Mobility Shift Assay (IP-EMSA)

HEK 293T cells were plated in p100 dishes at a density of 5×10^6^ and transfected with pcDNA3.1(+), pcDNA-MEIS1a-FLAG, pcDNA-MEIS2b-FLAG or pcDNA-MEIS3-FLAG using Lipofectamine 2000 according to the manufacturer's instructions. Forty-eight hrs. post transfection cells were washed twice with PBS. Eight hundred µl lysis buffer (50 mM TRIS pH 8.0, 150 mM NaCl, 1% Nonidet-P 40) containing protease inhibitors (Roche, Indianapolis, IN) were added subsequently. Cells were scraped, transferred to a micro-centrifuge tube and allowed to lyse on ice for 10 min. After clearing by centrifugation (10 min, 14000 rpm, 4°C), protein concentration was determined by the Bradford assay. 1.5 mg protein in a total volume of 500 µl were pre-cleared for 1 h at 4°C by adding 60 µl of a 50% slurry of washed protein agarose G beads (Invitrogen, Carlsbad, CA). Pre-cleared lysates were then incubated overnight at 4°C with 3 µg anti-FLAG (Sigma, St. Louis, MO) or 3 µg control non-immunogenic mouse IgG (Invitrogen, Carlsbad, CA). Samples were prepared for EMSA as described earlier [26]. Briefly, the immunoprecipitates were collected by centrifugation at 3,500 rpm for 1 min at 4°C and washed five times with wash buffer (20 mM HEPES, pH 7.9, 100 mM KCl, 0.2 mM EDTA, 20% glycerol), containing 1% NP-40 and once with wash buffer alone. Elution from beads was performed with 100 µl of 1.6% sodium deoxycholate in wash buffer for 15 min on ice. The sodium deoxycholate was neutralized with 12 µl of 10% NP-40, and the supernatant was directly used for EMSA. 20 µl of the supernatant were used for western blot analysis to confirm the efficiency of the IP.

### EMSA

80 fmol of γ^32^P labeled oligonucelotide were incubated with the supernatant for 20 min on ice in the presence of 1 µg of anti-FLAG, anti-MEIS1/2/3 (Millipore, Billerica, MA) or mouse control IgG in a reaction mix containing 5% glycerol, 50 mM KCl, 1 mM EDTA, 10 mM TRIS, 50 ng/ml poly(dI-dC) and 1 mM DTT. Complexes were resolved on a 5% polyacrylamid gel in 0.5× TBE buffer for 90 min at 200 V. The gels were then dried and exposed on X-ray film at −80°C. The *Krt19* promoter fragment used in this assay was 5′-CTAAGGGTGTCAAATTCCTG-3′.

### Western Blot analysis

Cells were lysed in IP buffer and protein concentration was normalized using Bradford reagent (Biorad, Hercules, CA). 25 µg were resolved on 4–12% Bis-Tris gels (Invitrogen, Carlsbad, CA), and transferred to polyvinylidene difluoride membranes (PVDF) as described. Membranes were blocked in either PBS containing 0.05% Tween and 5% non-fat dry milk or in blocking buffer (LiCor, Lincoln, NE) for 1 h at room temperature and incubated with one of the following antibodies at 4°C overnight: anti-FLAG (Sigma, St. Louis, MO) 1∶1000, anti-V5 (Invitrogen, Carlsbad, CA) 1∶5000, anti-MEIS1/2/3, clone 9.2.7 (Millipore, Billerica, MA) 1∶1000, anti-β-actin (Sigma, St. Louis, MO) 1∶5000, anti-PDX-1 A-17 (Santa Cruz Biotechnologies, Santa Cruz, CA) 1∶500, anti-Cip/Waf (BD Biosciences, Franklin Lakes, NJ) 1∶1000. Visualization was performed with either the appropriate HRP-coupled secondary antibody (GE Healthcare, Piscataway, NJ) and Western Lightning ECL reagents (Perking Elmer, Waltham, MA) on Hyperfilm ECL (GE Healthcare) or with the Odyssey Infrared Imaging System using IRDye 680 secondary antibodies (LiCor, Lincoln, NE).

### Immunoprecipitation

HEK 293T were transfected with pCMV-HA-Ubiquitin, pCDNA-MEIS1a-FLAG and pCMX-PDX-1 in equal amounts. Forty-eight hours post transfection cells were treated with either DMSO or Bortezomib (1 µM). IP and SDS-PAGE were essentially performed as described previously [20]. Briefly, cells were lysed in 800 µl lysis buffer (50 mM TRIS pH 8.0, 150 mM NaCl, 1% Nonidet-P 40) containing protease inhibitors (Roche, Indianapolis, IN). 1.5 mg protein in a total volume of 500 µl was pre-cleared for 1 h at 4°C by adding 60 µl of a 50% slurry of washed protein agarose beads (Invitrogen, Carlsbad, CA). Protein A agarose was used for antibodies produced in rabbit, Protein G agarose was used for all other antibodies. Pre-cleared lysates were then incubated overnight at 4°C with one of the following antibodies: anti-V5, anti-HA (Santa Cruz Biotechnologies, Santa Cruz, CA) or anti-FLAG. Proteins were separated by SDS-PAGE and detected by appropriate HRP-coupled secondary antibodies.

### Immunofluorescence staining

Eight to ten week old C/BL6 mice were sacrificed and the pancreas was removed quickly and fixed overnight in 4% para-formaldehyde. Organs were then embedded in paraffin, sectioned at 4 µm and mounted on glass slides. Following standard dewaxing and dehydration procedures, antigen retrieval was performed by microwaving the sections for 12 minutes at 600 W in buffered citric acid (pH 6.0). After permeabilization and blocking, sections were incubated overnight with the following antidodies: Keratin 19 (TROMA III, University of Iowa), MEIS1 (Abcam, Cambridge, MA, ab19867) and/or PDX-1 A-17 (Santa Cruz Biotechnologies, Santa Cruz, CA). Visualization was performed by incubation with Cy2 or Cy3 labeled appropriate secondary antibodies (Jackson Immunoresearch, West Grove, PA, 1∶600). Nuclear staining was achieved using 4′,6-Diamidino-2-phenylindole dihydrochloride (DAPI) (Sigma, St. Louis, MO). Images were obtained using a Nikon E 600 microscope (Nikon Instruments, Inc., Melville, NY) equipped with a Qicam Fast 1394 camera (Qimaging, Surrey, Canada) and iVision software (BioVision Technologies, Exton, PA).

### Statistics

A Student's t-test was used for simple comparisons. Analysis of variance (ANOVA) and Dunnett's post-test were performed in experiments that involved multiple comparisons. GraphPad Prizm Software built in analysis tools were used for all statistical analyses. A p<0.05 was considered statistically significant.

## Results

### PDX-1 represses *Krt19* reporter gene activation through the NH_2_-terminus

**Figure 1:**
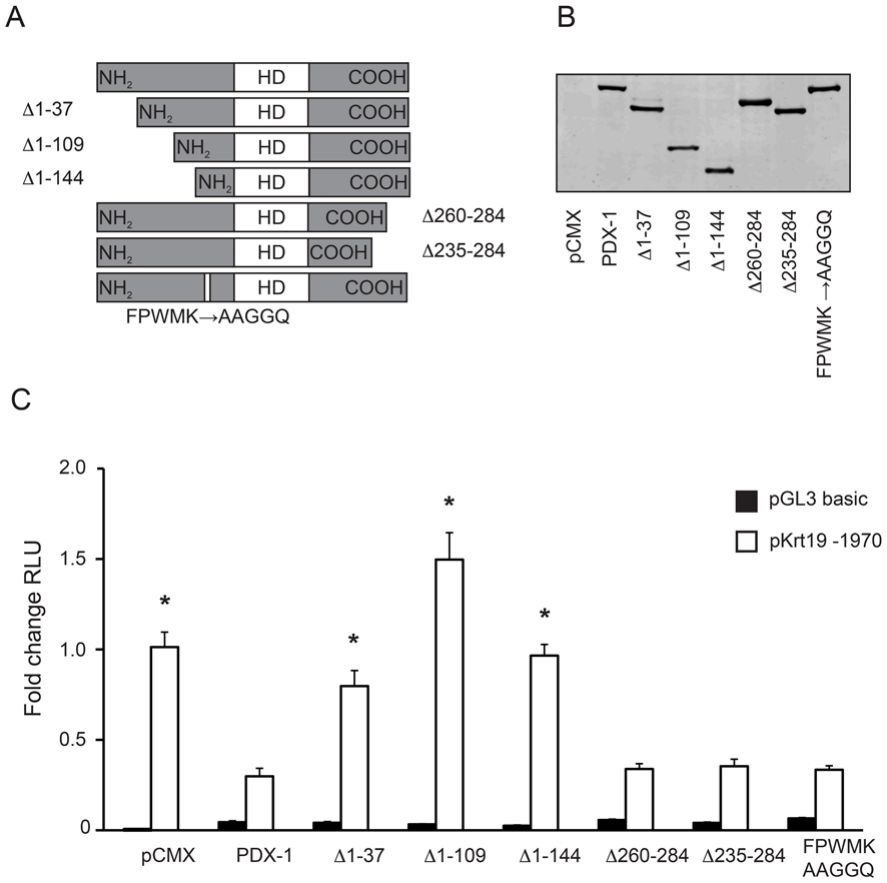
PDX-1 represses *Krt19* reporter gene activation through its NH_2_-terminus. A) Schematic overview of PDX-1 deletions and mutations. B) Western blot using an antibody against the V5-epitope demonstrated equal expression of PDX-1 deletions/mutations C) PDCs were transfected with pGL3 basic or pKrt19 -1970 reporter constructs and PDX-1 constructs shown in A). Reporter gene activity in cells transfected with empty vector was set arbitrarily to 1.0. NH_2_ terminal deletion, but not COOH terminal deletion or FPWMK to AAGGQ mutation, abrogates *Krt19* reporter gene repression. * p<0.05 as compared to PDX-1, significance was calculated by ANOVA and Dunnett's multiple comparison tests.

We assessed the pKrt19-1970 reporter gene activation by co-transfection of pancreatic ductal cells (PDC) with empty vector or full length PDX-1. *Krt19* reporter gene activation was reduced by 74.9±2.1% (p<0.05) in the presence of PDX-1 as compared to mock transfection (Fig. S1). These data are in line with previous findings [9]. To characterize the PDX-1 domains required for *Krt19* repression we generated PDX-1 mutants with deletions of the NH_2_- terminus (Δ1–37, Δ1–109 and Δ1–144), deletions of the COOH-terminus (Δ260–284 and Δ235–284) and an internal mutation of the amino acid sequence FPWMK to AAGGQ, thereby abrogating possible interaction of PDX-1 with PBX proteins in the latter PDX-1 mutant (Fig. 1A). Proper expression of all constructs was confirmed by western blot analysis (Fig. 1B). Deletion of the NH_2_-terminus resulted in significant relief of *Krt19* repression by PDX-1. Interestingly, the deletion of the very first 37 amino acids is sufficient for the relief of repression, whereas deletion of the COOH-terminus or mutation of the PBX interaction site had no effect on *Krt19* repression (Fig. 1B).

### The NH_2_-terminus of PDX-1 is important for binding to the *Krt19* promoter

**Figure 2:**
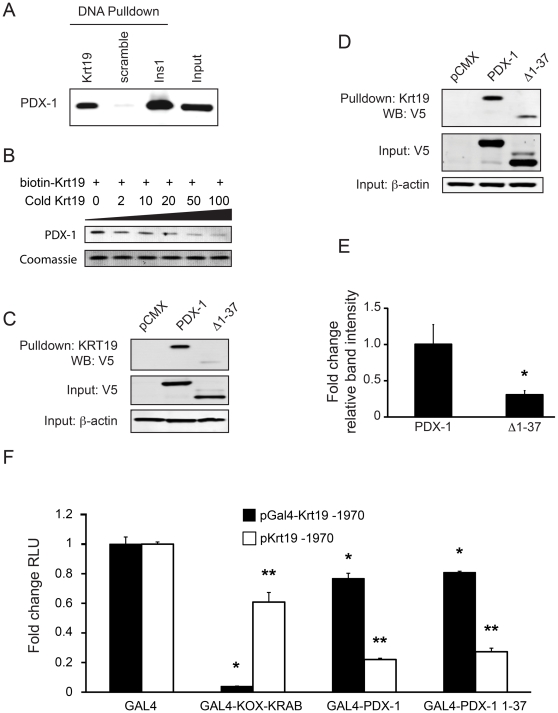
The NH_2_-terminus of PDX-1 binds to *Krt19* promoter DNA. A) MIN6 cells expressing endogenous PDX-1 were lysed and incubated with one of the following biotinylated DNA fragments: *Krt19*-promoter, scrambled negative control DNA or insulin promoter (*Ins1*, positive control), followed by precipitation with streptavidin agarose. Precipitated proteins and 2.5% input were separated with SDS-PAGE and blotted onto PVDF membranes. Membranes were probed with anti-PDX-1. PDX-1 specifically bound to *Krt19* and Insulin promoter DNA, but not scrambled negative control DNA. B) Extracts of MIN6 cells were incubated with 2 µg of biotinylated *Krt19* oligonucleotide and cold unbiotinylated *Krt19* oligonucleotide in increasing amounts as indicated [µg]. Separation of precipitated proteins was achieved by SDS-PAGE. Specifically precipitated PDX-1 was detected using anti-PDX-1, non-specifically precipitated proteins served as a loading control and were visualized by Coomassie staining. C) MIN6 cells were transfected with V5-tagged full length PDX-1 or PDX-1 Δ1-37. DNA precipitation was carried out as described above, and membranes were probed with anti-V5. Deletion of the NH_2_-terminus of PDX-1 resulted in an almost complete loss of PDX-1 binding to the *Krt19* promoter. D) PDX-1 negative HEK 293T cells were transfected with V5-tagged full length PDX-1 or PDX-1 Δ1–37. DNA precipitation was carried out as described above, and membranes were probed with anti-V5. PDX-1 Δ1–37 bound far less to the *Krt19* promoter fragment. E) Quantification of DNA precipitation assays in HEK 293T cells transfected with V5-tagged full length PDX-1 or PDX-1 Δ1–37. Band intensities were normalized to input levels prior to statistical analysis. Deletion of the NH_2_-terminus resulted in a significant decrease of PDX-1 binding to the *Krt19* promoter DNA. *p<0.05, Student t-test. F) PDCs were transfected with either pGal4-Krt19 -1970 or pKrt19 -1970 and pGAL-KOX-KRAB, pGAL-PDX-1 or pGAL-PDX-1 1-37. Reporter gene activity in cells transfected with empty vector was arbitrarily set to 1.0. GAL4-PDX-1 and GAL4-PDX-1 1–37 repressed pGal4-Krt19 -1970 slightly and pKrt19 -1970 profoundly. *p<0.05 for samples transfected with pGal4-Krt19 -1970; **p<0.05 for samples transfected with pKrt19 -1970. Significance was calculated by ANOVA and Dunnett's multiple comparison tests.

To confirm binding of endogenous PDX-1 to *Krt19* promoter DNA, we performed DNA precipitation assays in PDX-1 expressing MIN6 cells employing a *Krt19* promoter fragment that has been previously shown to be relevant for *Krt19* reporter gene repression [9]. Using this DNA pulldown assay, we were able to demonstrate interaction of endogenous PDX-1 with the *Krt19* promoter. Binding of PDX-1 to *Krt19* promoter DNA was specific as shown by the complete absence of a signal in the scrambled duplex control (Fig. 2A). As an additional proof of specificity we performed a competition assay using a cold (unbiotinylated) *Krt19* promoter fragment. Indeed, adding cold *Krt19* olilgonucleotide in increasing concentrations, as indicated, decreased binding of PDX-1 to the biotin-labeled probe, confirming the specificity of the assay (Fig. 2B). Next, we addressed the question whether binding of PDX-1 to *Krt19* promoter DNA is reduced upon deletion of the NH_2_-terminus. To that end, MIN6 cells were transfected with full length and PDX-1 Δ1–37. We found markedly decreased binding of PDX-1 Δ1–37 to the *Krt19* promoter fragment as compared to the full length PDX-1, suggesting that interaction of PDX-1 with the *Krt19* promoter requires the NH_2_-terminus of PDX-1 (Fig. 2C). To rule out possible competition of endogenous full length PDX-1 with exogenous Δ1–37 PDX-1 in this system, full length and Δ1–37 PDX-1 were transfected in HEK 293T cells, which do not express PDX-1. As expected, PDX-1 Δ1–37 displayed significantly reduced binding to the *Krt19* promoter (69.5±5.6% (p<0.05, Figs. 2D, E).

To further investigate if the amino acids (AA) 1–37 of PDX-1 have the ability to serve as a portable repressor domain, we fused full length PDX-1 and AA 1–37 to the DNA binding domain of GAL4 and performed co-transfection experiments in PDCs using a luciferase based reporter assay where the reporter is driven by a thymidine kinase promoter containing a 5xGal4 motif. Using this approach, we found that both full length PDX-1 and AA 1–37 increased reporter gene activation (data not shown). These observations are in line with PDX-1 being an activator of multiple genes, i.e. insulin or somatostatin and, more importantly, the fact that the NH_2_-terminus of PDX-1 serves as a transactivating domain on these target genes [27], [28]. Interestingly, gene activation through PDX-1 has been reported to require interaction of co-activators like p300 with the NH_2_-terminus of PDX-1 [29]. These data led to the question if there is a potentially sequence specific function of the PDX-1 NH_2_-terminus that mediates the regulation of *Krt19* transcription. To that end, we tested whether GAL4-PDX-1 and GAL4-PDX-1 1–37 have an impact on the activities of the wt-*Krt19* promoter and a mutant version that harbors a 5xGal4 motif. Surprisingly, both GAL4-PDX-1 and GAL4-PDX-1 1–37 slightly, but still significantly, repressed Gal4-*Krt19* transcriptional activation (Fig. 2F), although to a far lesser extent than the established repressor GAL4-KOX-KRAB [21], [30]. However, GAL4-PDX-1 and GAL4-PDX-1 1–37 were able to repress efficiently wt-*Krt19* transcriptional activation (Fig. 2F), whereas GAL4-KOX-KRAB was less effective. These data suggest that targeting PDX-1 directly to *Krt19* DNA impairs PDX-1's repressive function on transcriptional activation. By contrast, when PDX-1 is allowed to choose freely the site of interaction and possible interaction partners, it represses *Krt19* transcriptional activation. Most importantly this is also true for the amino acids 1–37 of PDX-1. Taken together, these data support the premise that there is a DNA sequence specific repressive function of the PDX-1 NH_2_-terminus, rather than a universal portable repressor domain.

### MEIS proteins bind to *Krt19* promoter region in an isoform-specific fashion

**Figure 3:**
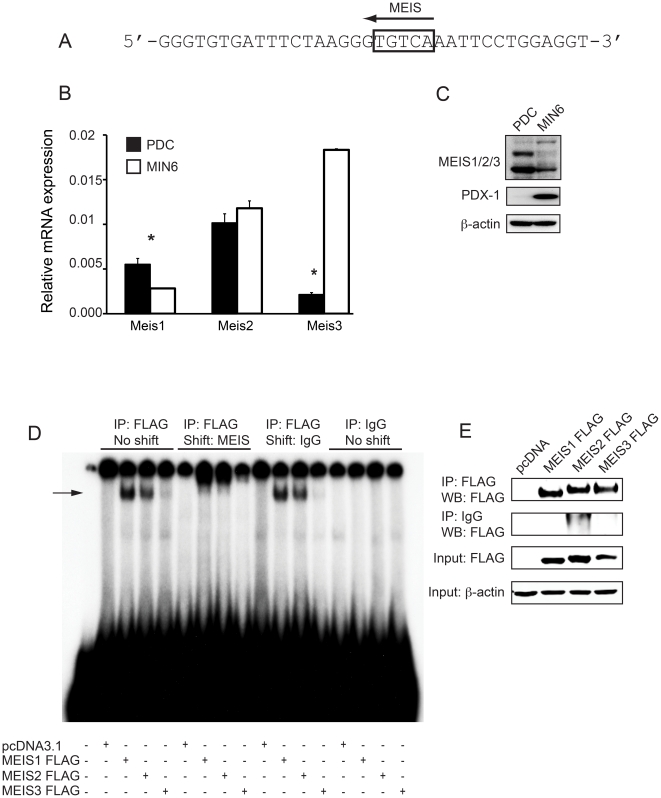
MEIS binds to the *Krt19* promoter in a homolog-specific fashion. A) Identification of a MEIS binding element in the *Krt19* promoter fragment. B) Quantitative Real-Time PCR was performed to detect *Meis* specific expression in PDX-1 negative PDCs and PDX-1 expressing MIN6 cells. *Meis1* and *Meis3* show significant differential expression in both cell lines with higher expression of *Meis1* in PDCs and higher levels of *Meis3* in MIN6 cells, whereas *Meis2* is expressed in equal amounts *p<0.05, Student's t-test. C) Lysates obtained from PDCs and MIN6 cells were separated by SDS-PAGE and transferred to PVDF membranes, which were probed with anti-MEIS1/2/3 antibody, anti-PDX-1 or anti-β-actin. Western blot analysis revealed differential expression of MEIS proteins in these two cell lines. D) HEK 293T cells were transfected with FLAG-tagged MEIS1, MEIS2 or MEIS3. Cells were lysed and immunoprecipitation using anti-FLAG was carried out. Precipitates were used for EMSA. IP against FLAG showed strong occupancy on the *Krt19* promoter fragment for MEIS1 and to a lesser extent for MEIS2, but not for MEIS3. Pre-incubation with anti-MEIS1/2/3 resulted in a shift for MEIS1 and MEIS2. Control IP with IgG did not yield a band. E) 20 µl of the input used for EMSA were separated by SDS-PAGE and transferred to PVDF membrane, followed by incubation with anti-FLAG. Western blot analysis showed equal amount of input and efficient pulldown for each FLAG-tagged MEIS protein.

We further investigated the mechanism by which PDX-1 may bind to the *Krt19* promoter. Although we were unable to identify a consensus TAA(T/T)TAT sequence for the binding of PDX-1 [31] the *Krt19* fragment used for precipitation assays harbors a MEIS binding site (Fig. 3A). This raised the question of whether *Krt19* expressing PDCs show different levels of MEIS proteins than *Krt19* negative PDX-1 positive endocrine MIN6 cells, and also whether there is differential expression of *Meis* genes. We first assessed mRNA levels of all three *Meis* genes in PDCs and MIN6 cells (Fig. 3B). We found significant differences in the mRNA levels of *Meis1*, having a significantly higher expression in PDCs, and *Meis3*, displaying a significantly higher expression in MIN6. *Meis2* mRNA is equally expressed in both cell types. Second, we evaluated expression of MEIS proteins by western blot (Fig. 3C). PDX-1 negative PDCs show higher expression of MEIS proteins, whereas PDX-1 positive MIN6 cells have overall lower levels of MEIS expression. Additionally, both cell lines express different MEIS proteins in line with the mRNA levels.

Due to the high sequence homology between MEIS proteins, similar molecular masses and different splice variants, it is difficult to precisely conclude which MEIS protein is detected by the antibody that recognizes all three isoforms. Therefore, we generated FLAG-tagged isoforms MEIS1a, MEIS2b and MEIS3 and transfected HEK 293T cells with either empty vector or plasmids coding for each of the MEIS proteins. Immunoprecipitations were performed and eluted complexes were incubated with radioactively labeled *Krt19* promoter fragment as described in the methods section. In this experiment, binding of MEIS1a and to a lesser extent MEIS2b, but not MEIS3, to the *Krt19* promoter fragment was revealed (Fig. 3D). To validate this approach, a supershift with anti-MEIS1/2/3 was performed. Co-incubation of the FLAG-immunoprecipitates with anti-MEIS1/2/3 resulted in a complete shift of MEIS1a and MEIS2b, whereas co-incubation with non-immunogenic control IgG did not alter the mobility of the complexes generated by MEIS1a and MEIS2b. The specificity of the IP was demonstrated by the absence of a signal when the IP was performed with IgG. To rule out possible variations caused by different efficiencies of the IP, a fraction of the IP was separated by SDS-PAGE and the membrane was probed with anti-FLAG antibody (Fig. 3E). The result demonstrated equal efficiencies of transfection and IP, suggesting specific DNA binding of MEIS1a and MEIS2b, but not MEIS3 (Fig. 3D). Noteworthy, if EMSA was performed directly with lysates of transfected cells, a shift could only be observed for MEIS1a, but not MEIS2b, suggesting that MEIS1a is the major MEIS protein that binds to the *Krt19* promoter. (Fig. S3).

### PDX-1 causes posttranscriptional down-regulation of MEIS1a

**Figure 4:**
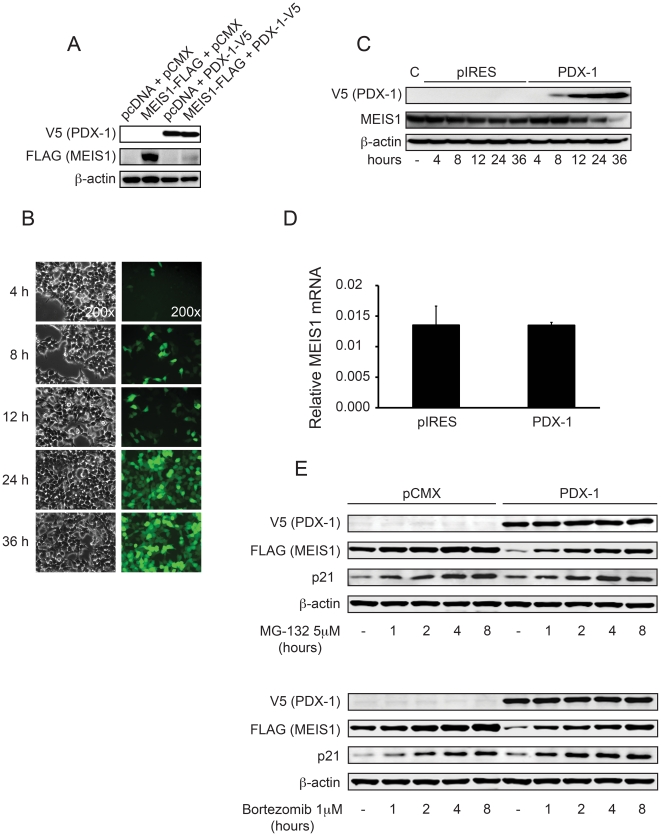
MEIS1 is regulated posttranscriptionally by PDX-1. A) HEK 293T cells were transfected with either MEIS1 or PDX-1 individually or in combination. Cells were lysed, and expression of PDX-1 and MEIS1 was evaluated by western blot analysis. Co-expression of PDX-1 and MEIS1 resulted in the absence of MEIS1. B) HEK 293T cells were co-transfected with pIRES2-EGFP-PDX-1 to assess the time course of expression after transfection by EGFP. Brightfield and matching fluorescence images are shown at 200× magnification. C) HEK 293T cells were transfected with pIRES2-EGFP-PDX-1 and lysates were harvested at the indicated time points. Western blot analysis revealed down-regulation of endogenous MEIS1 in a PDX-1 dependent manner. D) RNA was isolated 36 hrs. post transfection from HEK 293T cells that were transfected with PDX-1. Quantitative Real-Time PCR demonstrated stable mRNA levels of endogenous MEIS1. E) HEK 293T cells were transfected with MEIS1 and either empty vector control or PDX-1 and treated with DMSO or MG-132 or Bortezomib, respectively. Cells were lysed at indicated time points and subjected to western blot analysis. Both, MG-132 and Bortezomib were able to stabilize MEIS1 protein levels in the presence of PDX-1. p21 was unaffected by PDX-1 and served as a positive control for the functionality of MG-132 and Bortezomib, respectively.

Since homeobox proteins are capable of interacting with members of the TALE homeodomain protein family, we evaluated for possible protein-protein interactions between PDX-1 and MEIS1a by co-transfection of HEK 293T cells. Surprisingly, co-transfection resulted in absence of exogenous MEIS1a (Fig. 4A). For visualization of expression kinetics PDX-1 was expressed from a vector that co-expresses EGFP (Fig. 4B). Expression was observed as early as 4 hrs. post transfection and peaked at 24 hrs. post transfection. To rule out possible promoter competition between expression vectors we evaluated PDX-1 expression and endogenous MEIS1 expression at the indicated time points. Indeed, endogenous MEIS1 was down-regulated with increasing amounts of PDX-1 protein levels (Fig. 4C). To further elucidate the nature of MEIS1 down-regulation, *Meis1* mRNA levels were measured by quantitative RT-PCR 36 hours post transfection. *Meis1* mRNA levels were not changed in the presence of PDX-1, suggesting a possible posttranscriptional mechanism for MEIS1 regulation by PDX-1 (Fig. 4D). One possible mechanism in this setting would be ubiquitination with proteasomal degradation of MEIS1. To test this hypothesis, HEK 293T cells were co-transfected with PDX-1 and MEIS1a and then treated with the proteasome inhibitors MG-132 or Bortezomib. As before, co-transfection of PDX-1 and MEIS1a led to down-regulation of MEIS1a. However, this could be prevented by treatment with MG-132 (Fig. 4E, upper panel) or Bortezomib (Fig. 4E, lower panel). Stabilization of MEIS1a occurred in a time dependent fashion, and efficiency of drug treatment was proven by stabilization of the proteasomal regulated cell cycle protein p21.

To test the possibility that MEIS1a is degraded through ubiquitination and subsequently the proteasome, we evaluated MEIS1a for increased ubiquitination in the presence of PDX-1 with or without inhibition of the proteasome. Surprisingly, we detected increased laddering for MEIS1a in the absence and the presence of PDX-1, although to a lesser extent when PDX-1 was present (Fig. S4A). We then performed co-immunoprecipitation experiments with PDX-1, HA-tagged ubiquitin and FLAG-tagged MEIS1a in order to validate these findings. Indeed, in the presence of a proteasome inhibitor, HA-ubiquitin and FLAG-MEIS1a interact in the absence of PDX-1. By contrast, in the presence of PDX-1, an interaction could not be observed (Fig. S4B). Taken together, these data suggest a mechanism where MEIS1a is degraded by the proteasome in the presence of PDX-1, and this may happen independently of ubiquitination. Interestingly, this increased turnover of MEIS1a by PDX-1 does not appear to be dependent upon the NH_2_-terminus of PDX-1 (Fig. S4C), thereby indicating functional differences of PDX-1 with regards to binding to the *Krt19* promoter and the down-regulation of MEIS1a.

### *Meis1*, but not *Meis2*, is critical for *Krt19* transcriptional activity *in vitro* and expressed in pancreatic ductal cells *in vivo*

**Figure 5:**
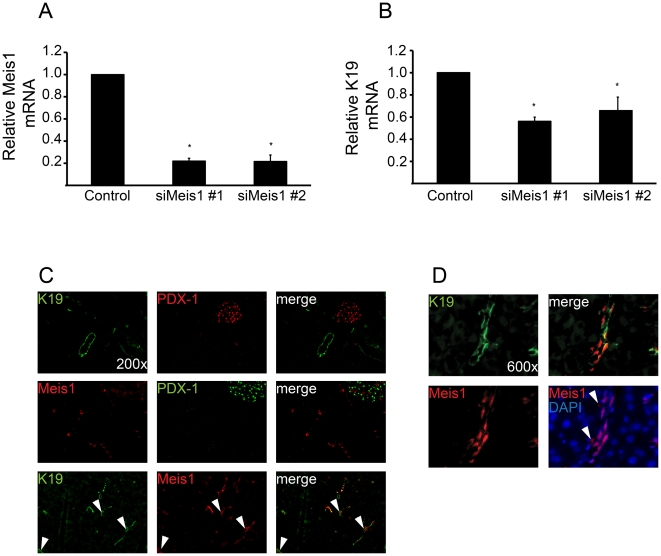
MEIS1 is essential for *Krt19* transcription *in vitro* and is expressed in pancreatic ducts *in vivo*. A) and B) PDCs were transfected with two different siRNAs against *Meis1* and RNA was isolated 72 hrs. post transfection. Genetic silencing of *Meis1* was efficient and resulted in a significant decrease of *Krt19* mRNA expression. *p<0.05. Statistical analysis was performed by ANOVA and Dunnett's multiple comparison test. C) Paraffin embedded mouse pancreatic tissue was stained for PDX-1, KRT19 and MEIS1 using a MEIS1 specific antibody. PDX-1 was expressed exclusively in islets, and there was no co-expression of MEIS1 and PDX-1. MEIS1 stained ducts expressing KRT19 and was found in a large proportion in the nuclei of ductal cells (arrowheads), whereas KRT19 was located in the cytoplasm (200× magnification). D) High power view of a pancreatic duct shows MEIS1 predominantly locates within the nuclei of ductal cells. Arrowheads indicate non-nuclear MEIS1 signals (600× magnification).

Finally, we wanted to know whether *Meis1* or *Meis2* are functionally relevant for *Krt19* transcrtiptional regulation. To address this question, *Meis1* and *Meis2* were genetically silenced in PDCs using two different siRNAs against *Meis1* or *Meis2* and RNA was extracted 72 h post transfection. Knockdown of *Meis1* and *Meis2* was efficient and similar for each siRNA used (Fig. 5A and S5A). Importantly, depletion of *Meis1* (Fig. 5B), but not *Meis2* (Fig. S5B), resulted in significantly reduced *Krt19* mRNA levels (56.11±3.74% and 65.77±11.82%, respectively) indicating that *Meis1* is directly involved in the transcriptional control of *Krt19* in pancreatic ductal cells. To evaluate this *in vivo*, we performed immunoflourescence staining on mouse pancreas for KRT19, PDX-1 and MEIS1 using a MEIS1-specific antibody. As expected, MEIS1 was expressed in pancreatic ducts together with KRT19, but not PDX-1 (Fig. 5C). Although there appeared to be some overlapping of KRT19 and MEIS1 (Fig. 5C), KRT19 staining was found in the cytoplasm, whereas MEIS localized predominantly in the nuclei of pancreatic ducts (Fig. 5D).

Taken together, our data indicate two mechanisms by which *Krt19* is regulated by PDX-1. The first one involves DNA binding of PDX-1 to the *Krt19* promoter that requires its NH_2_-terminus, and the second one acts through down-regulation of MEIS1, which is necessary for the maintenance of *Krt19* mRNA levels.

## Discussion

The goals of this study were to identify domains of PDX-1 necessary for mediation of *Krt19* transcriptional repression and to elucidate the functional interplay of PDX-1 with Hox-cofactors, namely MEIS. We created several NH_2_ terminal and COOH terminal deletions as well as an internal mutation of PDX-1. We found that the NH_2_-terminus, but not the COOH terminus or the pentapeptide motif, is critical in mediating *Krt19* transcriptional repression. The homeodomain, which was kept intact, harbors the DNA binding site of PDX-1 and also the nuclear translocation signal. Indeed, altered subcellular localization of PDX-1 does not occur upon NH_2_ terminal deletion (Fig. S2) and cannot account for the lack of repression of *Krt19* by these mutants. In particular, we demonstrated diminished binding of the PDX-1 NH_2_ terminal deletion to the promoter DNA of *Krt19*. Our data obtained with the GAL4 vector transfection studies support the premise that there is a DNA sequence specific repressive function of the PDX-1 NH_2_-terminus, rather than this region of PDX-1 serving as a universal portable repressor domain. Since direct binding of DNA by PDX-1 occurs via the homeodomain, it cannot be excluded that DNA binding mediated by the NH_2_-terminus is of an indirect nature, possibly through protein-protein interactions. This notion is supported by the fact that there is no canonical binding site for PDX-1 within the *Krt19* promoter.

Hox type transcription factors are known to bind to DNA either alone or in association with co-factors. This can happen as either heterodimeric or heterotrimeric complexes together with members of the TALE family, such as PBX and MEIS [32], [33]. PDX-1 has been reported to interact with PBX proteins via the FPWMK pentapeptide motif, and PBX in turn can recruit co-factors, such as histone deacetylases (HDACs), in order to mediate transcriptional repression [34].

We were able to exclude the involvement of PBX proteins in the repression of *Krt19* by PDX-1 through mutation of the PBX interaction motif as described previously [18]. Furthermore, treatment of cells with trichostatin A (TsA), an inhibitor of HDACs, does not alleviate the PDX-1 mediated repression of *Krt19* (data not shown). This is consistent with a previous report that the PDX-1 COOH-terminus is capable of recruiting HDACs to regulate gene transcription [35].

Since PBX proteins do not participate in the repression of *Krt19* by PDX-1 in a heterotrimeric complex consisting of PBX-PDX-1-MEIS, we explored the possibility of a MEIS-PDX-1 heterodimeric complex. We identified a MEIS binding site in the *Krt19* promoter and differential expression patterns of *Meis* genes with *Meis1* and *Meis2* being expressed in PDCs and *Meis2* and *Meis3* expression in MIN6 cells. We confirmed MEIS1 and MEIS2 as the major family members to bind the *Krt19* promoter. Interestingly, MEIS3, which displays only very low mRNA levels in PDCs, does not bind to the *Krt19* promoter. *Meis1* and Meis2 might cooperate to regulate vertebrate retina development through the maintenance of retinal progenitor cells [36]. That *Meis1* and *Meis2* may be more important for the exocrine ductal lineage is reinforced by the finding that the MEIS family members PREP1 and PREP2 form much stronger binding complexes with PBX1 on the *Pax6* pancreatic enhancer during islet cell development than do MEIS1 and MEIS2 [37].

To further understand the possible interaction between PDX-1 and MEIS1, we performed co-transfection experiments and found down-regulation of MEIS1 protein in the presence of PDX-1 while *Meis1* mRNA remained at a constant level. Additionally, we confirmed that the level of MEIS1 protein is inversely correlated with PDX-1 expression levels and MEIS1 levels are dependent directly on PDX-1 expression. For these reasons, we conclude that MEIS1 down-regulation occurs on a posttranscriptional or basis. One possible mechanism of how protein levels are regulated posttranscriptionally is by increased turnover and degradation by the proteasome. Indeed, MEIS1 protein could be stabilized in the presence of PDX-1 through inhibition of the proteasome. However, we were unable to demonstrate substantially increased ubiquitination of MEIS1 in the presence of PDX-1. One simple explanation might be that the overall level of MEIS1 is reduced when PDX-1 is present, which makes the detection of ubiquitinated MEIS1 difficult. Another possibility that needs to be explored further is that the degradation of MEIS1 through the proteasome is independent of ubquitination, an exciting mechanism that has become increasingly recognized [38] and has been described for important and well-studied transcription factors, such as c-Fos, p53 and p73 [39], [40], [41]. Taken together, our data suggest different ways of how PDX-1 mediates the transcription of *Krt19*. First, we provide evidence for direct repression through binding to the *Krt19* promoter, and second, PDX-1 fosters proteasomal degradation of MEIS1. Third, we have demonstrated that MEIS1 is a regulatory transcription factor that is necessary for *Krt19* transcriptional activation in PDCs. Since *Meis2* knockdown does not affect *Krt19* mRNA, we believe *Meis2* does not have a predominant role when compared to *Meis1*.

The regulation of MEIS1 is poorly understood, although there is evidence for transcriptional regulation by Creb [42]. Here, we show for the first time that MEIS1 appears to be posttranscriptionally regulated by a mechanism that requires PDX-1. One scenario might be the activation of an as yet unidentified MEIS1 interacting factor by PDX-1. We have shown previously that MEIS1 activates the *Krt19* promoter, and importantly, that this property could not overcome PDX-1 mediated repression of *Krt19* [9]. We now conclude that the posttranscriptional down-regulation of MEIS1, both endogenous and exogenous, is responsible for this effect.

**Figure 6:**
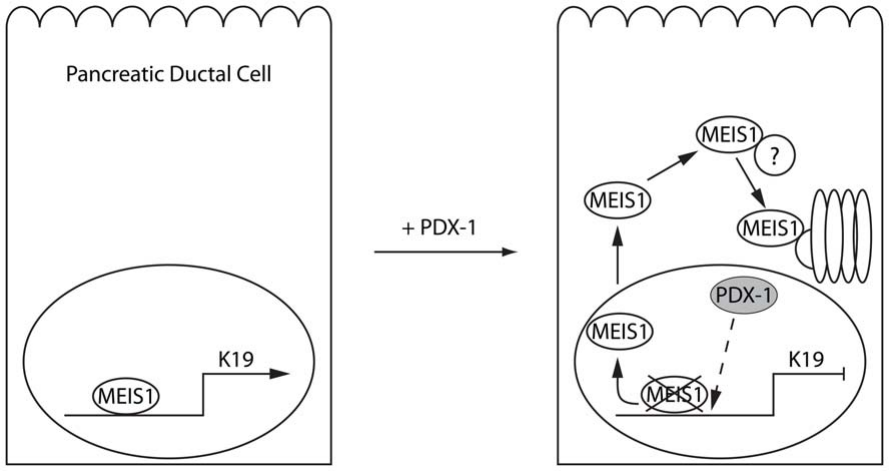
Proposed model of how PDX-1 regulates *Krt19* transcriptional activity. In a pancreatic duct cell, PDX-1 is not expressed and MEIS1 is able to occupy the *Krt19* promoter and transactivate *Krt19* transcription (left panel). By contrast, PDX-1 leads to displacement of MEIS1 from the *Krt19* promoter and its degradation by the proteasome through mechanisms not yet fully elucidated. PDX-1 in turn binds to the *Krt19* promoter via its NH_2_ terminal domain and consequently represses *Krt19* transcriptional activation (right panel).

A possible mechanism for up-regulation of MEIS target genes is suggested by the recent finding that MEIS proteins increase the histone H4 acetylation status on certain promoters during zebrafish embryogenesis [43]. In this context, MEIS acts by regulating the access of HDAC and CBP to such promoters. Consequently, loss of MEIS1 would result in decreased transcriptional activity of critical target genes. For example, zebrafish embryos lacking *Meis1* have a marked reduction in *gata1* expression, which is critical for erythroid cell fate, and lack circulating blood cells [44]. This can be observed in the direct regulation of the developmentally important SOX3 by MEIS1 and PBX [45]. Given the overall importance of PDX-1 during development and differentiation of the pancreas, it is tempting to speculate that continued expression of PDX-1 would lead to increased turnover of MEIS1 and prevent expression of *Krt19* in the endocrine lineage. Down-regulation of *Krt19* transcription may then be further secured by direct interference of PDX-1 with the *Krt19* promoter in the ductal lineage; indeed, PDX-1 is down-regulated or absent in differentiated ductal cells. Loss of PDX-1 could lead to stabilization of MEIS1, relief of repression at the *Krt19* promoter and subsequent ductal specific *Krt19* gene expression (Fig. 6). This model would complement the observation that regulated loss of PDX-1 at mid-pancreatic development may still lead to the formation of a truncated ductal tree, but not of acinar tissue [46].

## Supporting Information

Table S1 Sequences of primers that have been used for the generation of PDX-1 mutants and mutation of pKrt19-1970. Primer sequences were selected to generate the indicated truncation or mutation of PDX-1 of for fusion of PDX-1 and PDX-1 1-37 to the GAL4 DNA binding domain. The primer pKrt19 -1970 was used to introduce AgeI and SacII restricion sites into pKrt19 -1970. Sequences in lower case introduced the desired mutations.
(0.04 MB DOC)

Table S2 Primers for quantitative RT-PCR. Primers for quantitative RT-PCR were designed for the specific mRNA as indicated. Sequences were selected to be intron-spanning to avoid amplification of genomic DNA.
(0.03 MB DOC)

Figure S1 Expression of PDX-1 in PDCs reduces Krt19 transcriptional activation. PDCs were transfected with either pCMX or PDX-1 together with pGL3 basic or pKrt19-1970 luciferase reporter construct. Expression of PDX-1 resulted in significant decrease of the luciferase reporter activity.
(0.21 MB TIF)

Figure S2 Proper subcellular localization of PDX-1 is maintained upon NH_2_ terminal deletion. HEK 293T cells were transfected with either empty vector, PDX-1 or PDX-1 Δ 1-37 and stained with anti-V5 antibody. Deletion of amino acids 1–37 does not affect translocation of PDX-1 to the nucleus (600×).
(1.29 MB TIF)

Figure S3 MEIS1 binds to the *Krt19* promoter DNA. HEK 293T cells were transfected with FLAG-tagged MEIS proteins MEIS1, MEIS2 and MEIS3. A supershift was performed using anti-FLAG antibody. A bandshift was observed when Krt19 promoter DNA was incubated with lysates from MEIS1 expressing cells (arrow), but not from MEIS2 or MEIS3 expressing cells. Of note, the band obtained with lysates from MEIS2 expressing cells is distinct in size than the one observed with lysates from cells that expressed MEIS3 or control cells.
(3.47 MB TIF)

Figure S4 PDX-1 does not substantially increase ubiquitination of MEIS1. A) HEK 293T cells were co-transfected with empty vector or PDX-1 and MEIS1-FLAG and treated with DMSO or proteasome inhibitors as indicated for 8 hrs. Follwing SDS-PAGE and protein transfer to PVDF membranes, visualization was perfomed by HRP-coupled secondary antibodies. Long exposure (3 minutes) revealed markedly increased laddering of MEIS1 in mock transfected samples and slightly increased laddering in the presence of PDX-1 when samples were treated with proteasome inhibitors. p21 served as a control for the efficiency of the drugs. B) HEK 293T cells were transfected with equal amounts of pCMV-HA-ubiquitin, PDX-1 and MEIS1a-FLAG. IP revealed interaction of ubiqutin and MEIS1 in absence, but not in presence of PDX-1, when samples were treated with the proteasome inhibtor Bortezomib (1 µM). Notably, MEIS1 levels were reduced in samples co-transfected with PDX-1, either with or without inhibition of the proteasome as revealed by short exposure and input, respectively. C) The down-regulation of MEIS1 by PDX-1 occurs independently of the C-terminus of PDX-1.
(1.66 MB TIF)

Figure S5 *Meis2* is not required for the regulation of *Krt19*. PDCs were transfected with two different siRNAs against *Meis2* and RNA was isolated 72 hrs. post-transfection. A) Knockdown of *Meis2* using two independent siRNA was highly efficient. B) Despite sufficient knockdown of *Meis2*, *Krt19* mRNA levels did not change significantly. *p<0.05. Statistical analysis was performed by ANOVA and Dunnett's multiple comparison test.
(0.35 MB TIF)
