# Supplementary material for: The Pancreatic and Duodenal Homeobox Protein PDX-1 Regulates the Ductal Specific Keratin 19 through the Degradation of MEIS1 and DNA Binding
Source: PLoS One. 2010 Aug 19;5(8):e12311. doi: 10.1371/journal.pone.0012311 (PMC2924401; doi:10.1371/journal.pone.0012311)
Supplement: Table S1 — Sequences of primers that have been used for the generation of PDX-1 mutants and mutation of pKrt19-1970. Primer sequences were selected to generate the indicated truncation or mutation of PDX-1 of for fusion of PDX-1 and PDX-1 1-37 to the GAL4 DNA binding domain. The primer pKrt19 -1970 was used to introduce AgeI and SacII restricion sites into pKrt19 -1970. Sequences in lower case introduced the desired mutations. (0.04 MB DOC) [file pone.0012311.s001.doc]

Table S1

| **Primer** | **Sequence 5’→3’** |
| --- | --- |
| Pdx-1 FW | GCCACCATGAACAGTGAGGAGC |
| Pdx-1 RV | TCACGTAGAATCGAGACCGAGGAGAGGGTTAGGGATAGGCTTACCCCGGGGTTCCTGCGGT |
| Pdx1 ∆1-37 FW | GCCACCATGGGCCGCCAGCCC |
| Pdx1 ∆1-37 RV | See Pdx1 |
| Pdx1 ∆1-109 FW | GCCACCATGCTGGAAGAGCCCAACCGC |
| Pdx1 ∆1-109 RV | See Pdx1 |
| Pdx1 ∆1-144 FW | GCCACCATGGAGGAAAACAAGAGGACCCGTACT |
| Pdx1 ∆1-144 RV | See Pdx1 |
| Pdx1 ∆260-284 FW | See Pdx1 |
| Pdx1 ∆260-284 RV | TCACGTAGAATCGAGACCGAGGAGAGGGTTAGGGATAGGCTTACCCTCCCGGACTGCAGCTGG |
| Pdx1 ∆235-284 FW | See Pdx1 |
| Pdx1 ∆235-284 RV | TCACGTAGAATCGAGACCGAGGAGAGGGTTAGGGATAGGCTTACCCTCGCCCGAGGTCACCG |
| FPWMK→AAGGQ | CGCGTCCAGCTCCCTgcagcaggaggtcaaTCCACCAAAGCTCAC |
| Gal4Pdx1 FW | GTATGAACAGTGAGGAGCAGTACTACGC |
| Gal4Pdx1 RV | CTACCGGGGTTCCTGCGGT |
| Gal4Pdx1 1-37 FW | See Gal4Pdx1 |
| Gals4Pdx 1-37 RV | CTAGTACAGGCACGCAGGGGG |
| pKrt19 -1970 | GTAAGTCACTGAGTccgcggaccggtTGGGGCTCAGAGGG |
