# Supplementary material for: The Pancreatic and Duodenal Homeobox Protein PDX-1 Regulates the Ductal Specific Keratin 19 through the Degradation of MEIS1 and DNA Binding
Source: PLoS One. 2010 Aug 19;5(8):e12311. doi: 10.1371/journal.pone.0012311 (PMC2924401; doi:10.1371/journal.pone.0012311)
Supplement: Table S2 — Primers for quantitative RT-PCR. Primers for quantitative RT-PCR were designed for the specific mRNA as indicated. Sequences were selected to be intron-spanning to avoid amplification of genomic DNA. (0.03 MB DOC) [file pone.0012311.s002.doc]

Table S2

| **mRNA** | **FW Primer 5’→3’** | **RV Primer 5’→3’** |
| --- | --- | --- |
| *Meis1* | ATGACACGGCATCCACTCGTTC | TGTCCAAGCCATCACCTTGCT |
| *Meis2* | TGTCAACGACGCCTTGAAAAG | GCTCGCACTTCTCAAAAACCA |
| *Meis3* | GTGGCCACCAACATCATGA | CCAGCTGTTTCTTTTGCTCTTC |
| *Krt19* | TCCCAGCTCAGCATGAAAGCT | AAAACCGCTGATCACGCTCTG |
| *Cyclophilin A* | ATGGTCAACCCCACCGTGT | TTCTGCTGTCTTTGGAACTTTGTC |
| *MEIS1* | ATGACACGGCATCTACTCGTTC | TGTCCAAGCCATCACCTTGCT |
| *β-ACTIN* | CCTGGCACCCAGGACAAT | GCCGATCCACACGGAGTACT |
