# Supplementary figures and images for: The Pancreatic and Duodenal Homeobox Protein PDX-1 Regulates the Ductal Specific Keratin 19 through the Degradation of MEIS1 and DNA Binding
Source: PLoS One. 2010 Aug 19;5(8):e12311. doi: 10.1371/journal.pone.0012311 (PMC2924401; doi:10.1371/journal.pone.0012311)

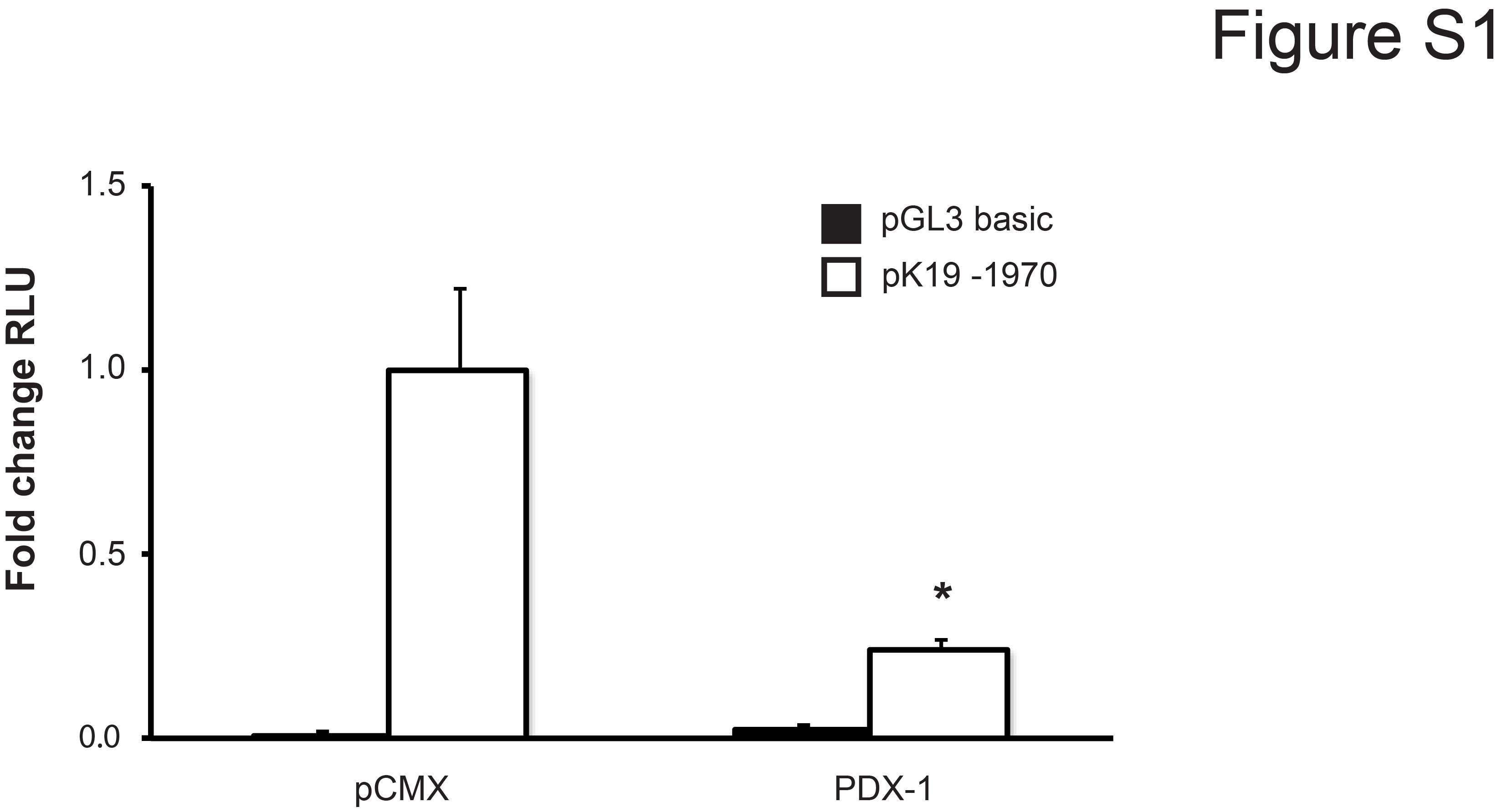

Supplement: Figure S1 — Expression of PDX-1 in PDCs reduces Krt19 transcriptional activation. PDCs were transfected with either pCMX or PDX-1 together with pGL3 basic or pKrt19-1970 luciferase reporter construct. Expression of PDX-1 resulted in significant decrease of the luciferase reporter activity. (0.21 MB TIF) [file pone.0012311.s003.tif]

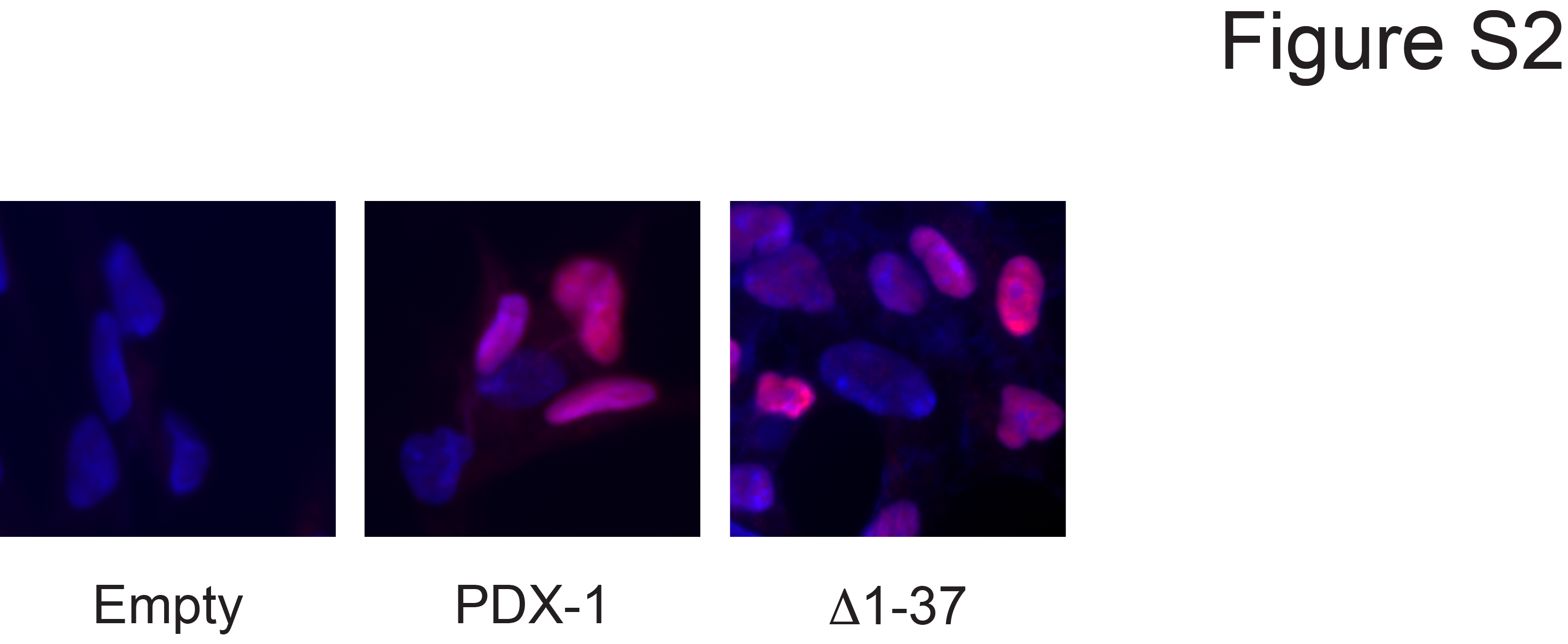

Supplement: Figure S2 — Proper subcellular localization of PDX-1 is maintained upon NH2 terminal deletion. HEK 293T cells were transfected with either empty vector, PDX-1 or PDX-1 Δ 1-37 and stained with anti-V5 antibody. Deletion of amino acids 1–37 does not affect translocation of PDX-1 to the nucleus (600×). (1.29 MB TIF) [file pone.0012311.s004.tif]

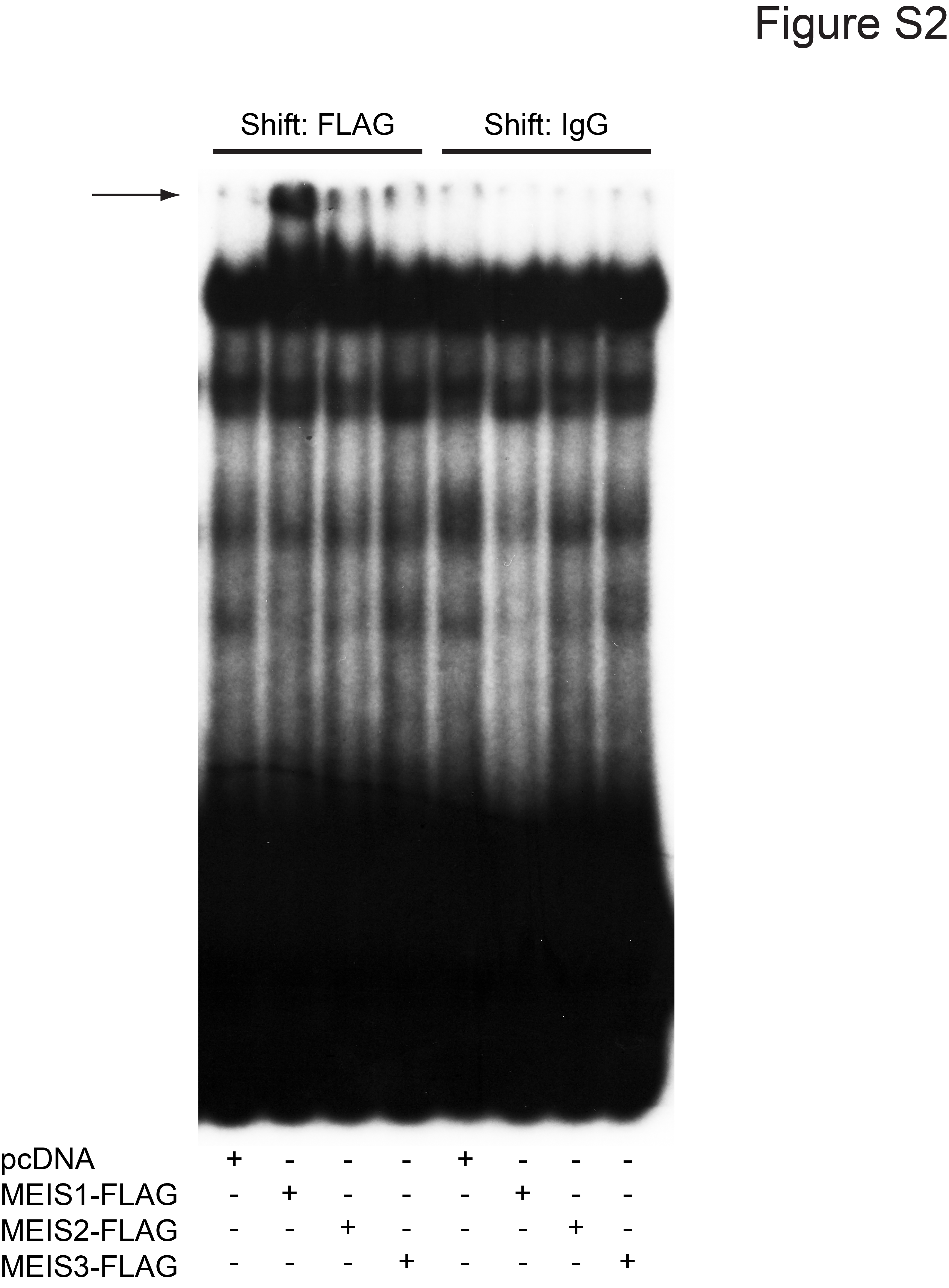

Supplement: Figure S3 — MEIS1 binds to the Krt19 promoter DNA. HEK 293T cells were transfected with FLAG-tagged MEIS proteins MEIS1, MEIS2 and MEIS3. A supershift was performed using anti-FLAG antibody. A bandshift was observed when Krt19 promoter DNA was incubated with lysates from MEIS1 expressing cells (arrow), but not from MEIS2 or MEIS3 expressing cells. Of note, the band obtained with lysates from MEIS2 expressing cells is distinct in size than the one observed with lysates from cells that expressed MEIS3 or control cells. (3.47 MB TIF) [file pone.0012311.s005.tif]

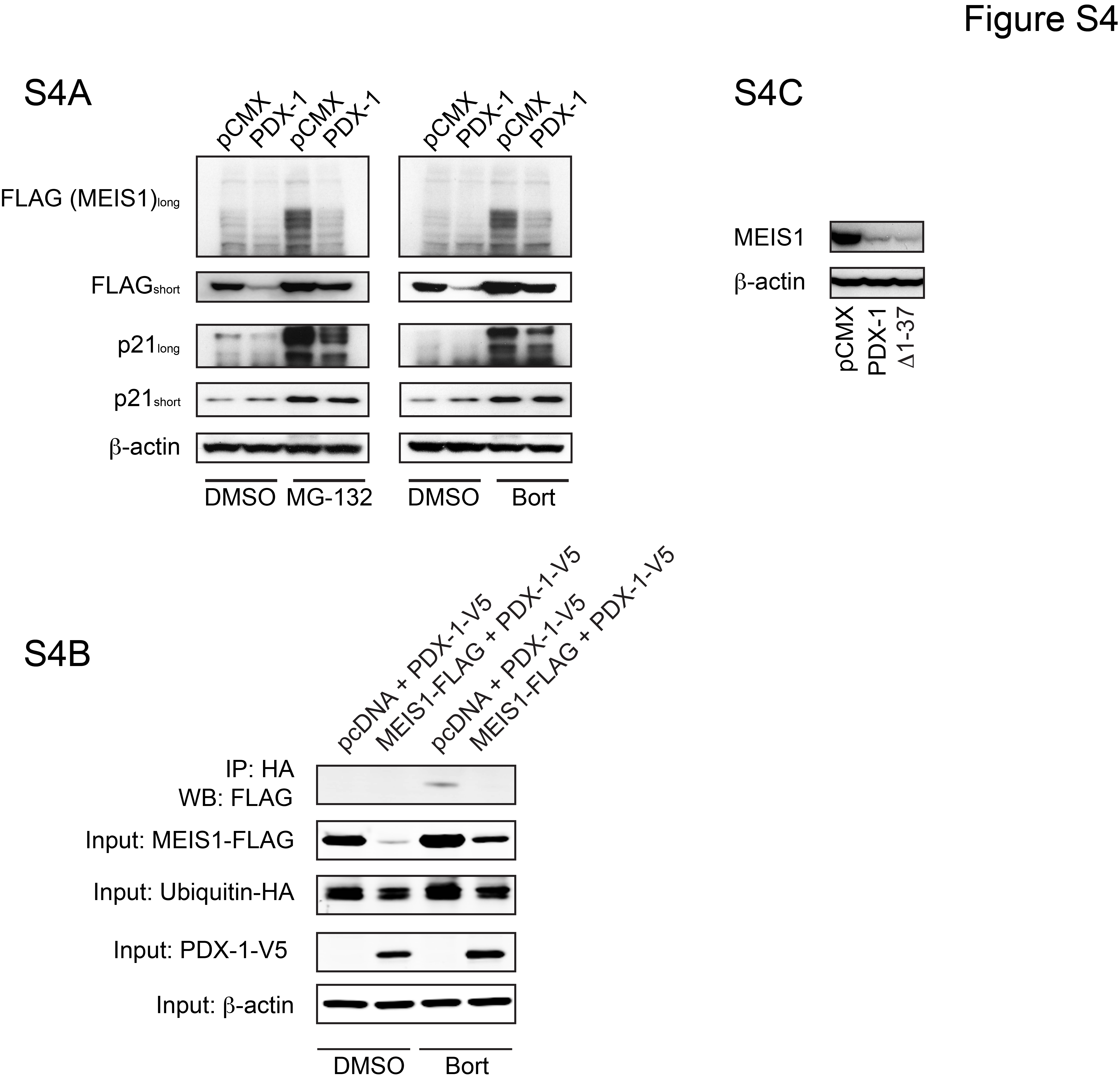

Supplement: Figure S4 — PDX-1 does not substantially increase ubiquitination of MEIS1. A) HEK 293T cells were co-transfected with empty vector or PDX-1 and MEIS1-FLAG and treated with DMSO or proteasome inhibitors as indicated for 8 hrs. Follwing SDS-PAGE and protein transfer to PVDF membranes, visualization was perfomed by HRP-coupled secondary antibodies. Long exposure (3 minutes) revealed markedly increased laddering of MEIS1 in mock transfected samples and slightly increased laddering in the presence of PDX-1 when samples were treated with proteasome inhibitors. p21 served as a control for the efficiency of the drugs. B) HEK 293T cells were transfected with equal amounts of pCMV-HA-ubiquitin, PDX-1 and MEIS1a-FLAG. IP revealed interaction of ubiqutin and MEIS1 in absence, but not in presence of PDX-1, when samples were treated with the proteasome inhibtor Bortezomib (1 µM). Notably, MEIS1 levels were reduced in samples co-transfected with PDX-1, either with or without inhibition of the proteasome as revealed by short exposure and input, respectively. C) The down-regulation of MEIS1 by PDX-1 occurs independently of the C-terminus of PDX-1. (1.66 MB TIF) [file pone.0012311.s006.tif]

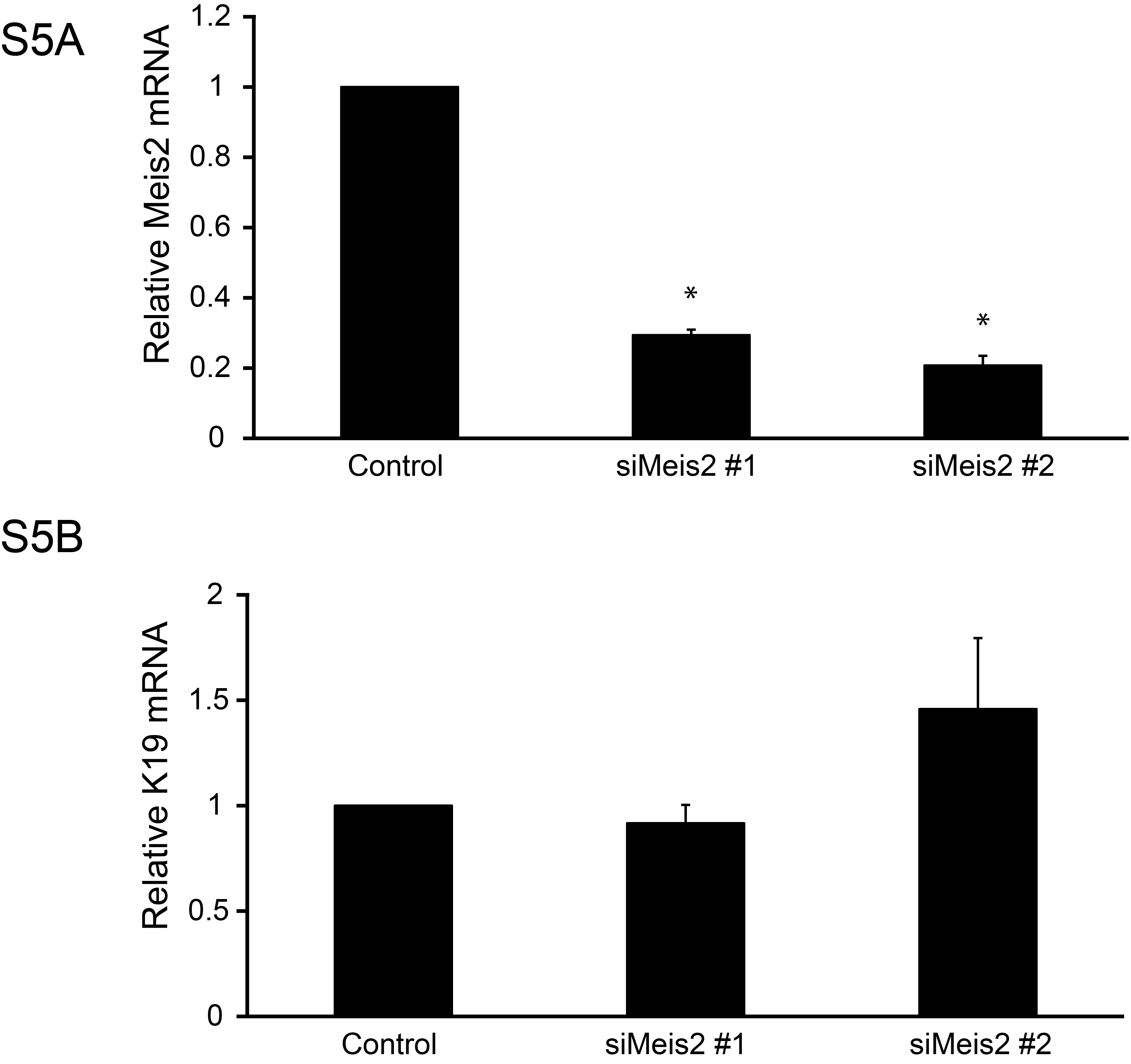

Supplement: Figure S5 — Meis2 is not required for the regulation of Krt19. PDCs were transfected with two different siRNAs against Meis2 and RNA was isolated 72 hrs. post-transfection. A) Knockdown of Meis2 using two independent siRNA was highly efficient. B) Despite sufficient knockdown of Meis2, Krt19 mRNA levels did not change significantly. *p<0.05. Statistical analysis was performed by ANOVA and Dunnett's multiple comparison test. (0.35 MB TIF) [file pone.0012311.s007.tif]
